# Iodoform in Surgical Practice: A Comprehensive Review of Its Historical Evolution, Clinical Applications, and Safety Profiles

**DOI:** 10.7759/cureus.75752

**Published:** 2024-12-15

**Authors:** Dia R Halalmeh, Yusuf-Zain Ansari, Arwa Jader, Husam Eddin Z Salama, Carmelo V Venero, Marc D Moisi

**Affiliations:** 1 Department of Neurosurgery, Hurley Medical Center, Flint, USA; 2 Department of Neurosurgery, University of Iowa Hospitals and Clinics, Iowa City, USA; 3 College of Science and Technology, Temple University, Philadelphia, USA; 4 Department of Neurosurgery, University of Kufa, Kufa, IRQ; 5 Department of Medicine and Surgery, Al-Quds University, Jerusalem, PSE; 6 Department of Neurosurgery, University of Kentucky College of Medicine, Lexington, USA

**Keywords:** antiseptic, bismuth iodoform paraffin paste (bipp), clinical applications, iodoform, neurosurgery, surgical packing

## Abstract

Iodoform, a halogenated organic compound, has been a cornerstone in surgical practice due to its potent antiseptic and antimicrobial properties. This comprehensive review examines the historical evolution, mechanism of action, clinical applications, and safety profile of iodoform across various surgical disciplines. Historically significant formulations like Whitehead's varnish and bismuth iodoform paraffin paste (BIPP) demonstrated remarkable efficacy in wound healing during the late 19th and early 20th centuries. BIPP, extensively used for cavity packing, combines bismuth subnitrate for its antibacterial and astringent effects with paraffin to minimize tissue trauma. The antimicrobial action of iodoform is attributed to the release of iodine upon activation by free radicals, leading to the denaturation of bacterial proteins and cytotoxic effects on inflammatory cells, thereby enhancing therapeutic efficacy. Clinically, iodoform has diverse applications in ear, nose, and throat surgery, neurosurgery, oral and maxillofacial surgery, and general dental practice. It is utilized for dressing wounds, packing surgical cavities, and managing conditions such as nasal fractures, epistaxis, cerebrospinal fluid leaks, and dry sockets. The radiopacity of BIPP, due to its bismuth content, aids in radiographic identification but necessitates clear communication with radiologists to prevent misinterpretation. Despite its benefits, iodoform use is associated with potential complications. Bismuth and iodoform toxicities, though rare, can lead to neurotoxicity and systemic symptoms, requiring prompt recognition and intervention. Allergic reactions, particularly in patients with prior exposure, and dermatological side effects like dermatitis herpetiformis flare-ups have been documented. Mechanical complications and considerations during pregnancy, owing to the potential transfer of iodine to the fetus, highlight the need for cautious application. This review underscores the enduring significance of iodoform in surgical settings while emphasizing the importance of awareness regarding its potential risks. Careful clinical judgment and ongoing research are imperative to optimize its therapeutic benefits and enhance patient safety.

## Introduction and background

Introduction and mechanism of action

Iodoform, also known as tri-iodomethane, is an organoiodine compound where three hydrogen atoms of methane are replaced by iodine atoms. Discovered by Georges Serullas in 1822, iodoform has a long-standing history in medical formulations due to its antimicrobial and antiseptic properties [1]. Chemically reactive in free radical reactions, iodoform exhibits antimicrobial efficacy when administered topically. The proposed mechanism involves activation by free radicals, leading to the release of iodine, which denatures bacterial proteins and effectively eliminates pathogenic microorganisms. At high concentrations, iodoform also exerts cytotoxic effects on inflammatory cells infiltrating the wound, destroying them and enhancing their therapeutic efficacy [2].

Over the years, various formulations containing iodoform have been developed and utilized in medical practice. Commonly, iodoform dressings are combined with agents like glycerine, eucalyptus oil, or bacitracin ointment to enhance their therapeutic effects. Notable formulations include Whitehead's varnish, bismuth iodoform paraffin paste (BIPP), Alvogyl, and Vitapex® (Neodental International) [1,3]. Whitehead’s varnish was introduced in 1891 by Walter Whitehead, incorporating iodoform as a key ingredient to leverage its antiseptic properties. By 1917, Rutherford Morison described BIPP in his work, reporting accelerated healing and reduced infection rates when applied to open wounds [4]. BIPP was notably used during World War I for treating gunshot wounds and has continued to be a valuable wound-packing material since then.

**Figure 1:**
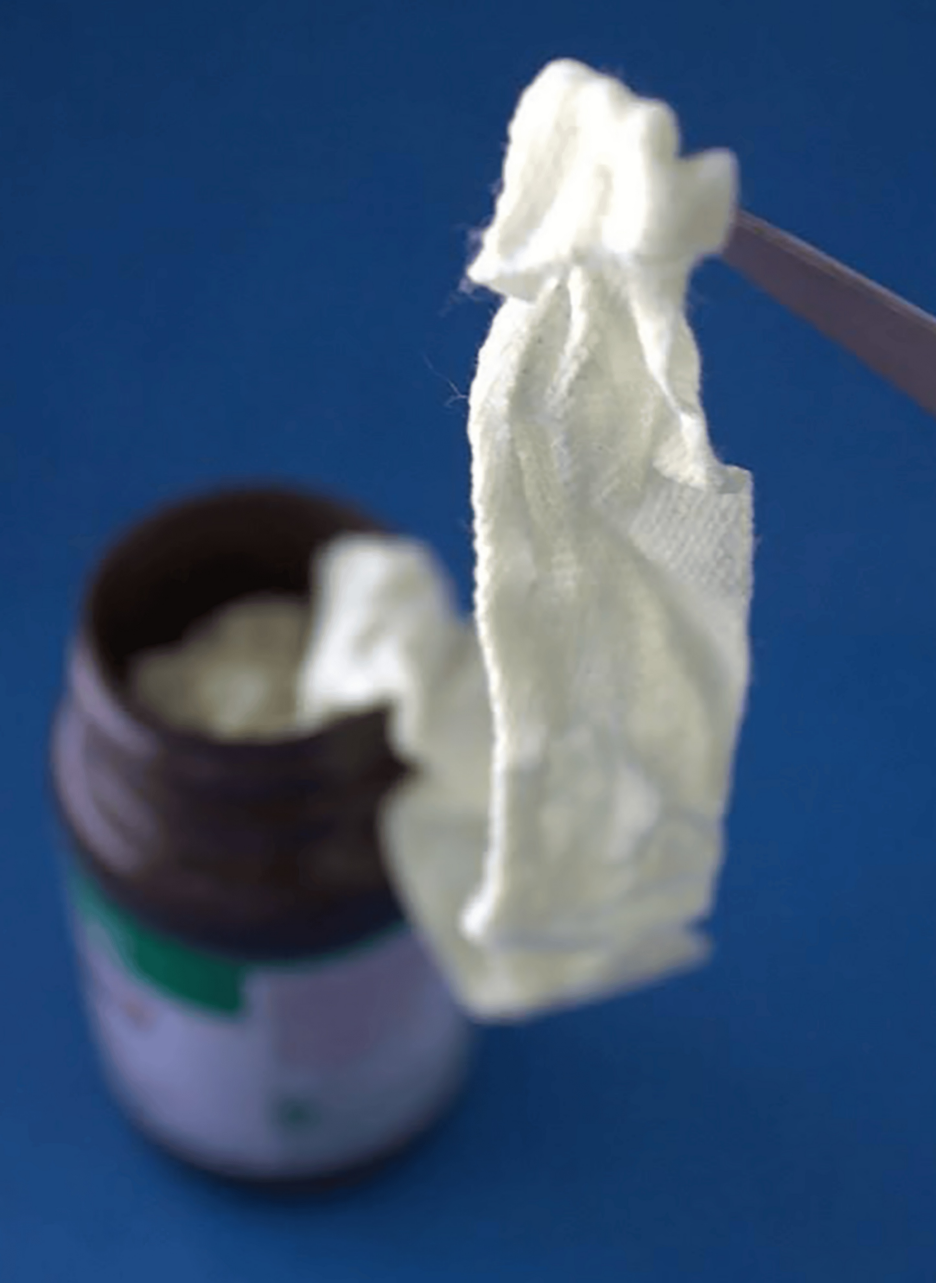
BIPP impregnated gauze BIPP: bismuth iodoform paraffin paste Image Credit: Kristin N. MacPheert

Currently, BIPP is the most commonly used form of iodoform, available as an impregnated gauze (Figure 1). The formulation includes bismuth subnitrate, which acts as an astringent to reduce tissue contraction and contributes to antibacterial properties, while paraffin serves as a lubricant to minimize tissue trauma during packing [1,5]. This combination facilitates seamless application and removal of BIPP. The antiseptic properties of BIPP are further enhanced by nitric acid, which is speculated to potentiate the release of iodine [6]. Iodoform has also been shown to be effective in chemical debridement by facilitating collagen fibrinolysis and the lysis of necrotic tissue [7]. This action mimics that of collagenases, essential enzymes for removing unprocessed collagen fibers that would otherwise anchor necrotic tissue to the wound surface. Additionally, iodoform can stimulate granulation tissue formation, thus promoting wound healing [8]. Besides its antiseptic properties, iodoform imparts marked anesthetic effects, and the impregnated gauze's impermeability to blood and body fluids makes it resistant to bacterial growth.

Various forms of iodoform have found applications across multiple medical fields, including dentistry, oral and maxillofacial surgery, otolaryngology, and general surgery. For instance, Alveogyl, a brown fibrous paste containing iodoform, is commonly used in dentistry for the treatment of dry sockets. The objective of this review is to consolidate the available literature into a comprehensive analysis, thereby improving knowledge about the history, efficacy, safety, and use of iodoform across multiple surgical fields.

Historical evolution

The synthesis of iodoform was first described in 1822 by Georges-Simon Serullas, a French professor of pharmacy, who produced it by reacting iodine vapor with steam over red-hot coals [7]. Recognized for its antiseptic and disinfectant properties, iodoform started to be incorporated into wound-healing formulations from the late 1800s to the early 1900s, marking its significance in surgical practices.

In 1891, Walter Whitehead introduced Whitehead’s varnish, a formulation renowned for its anesthetic and adhesive properties, in his paper titled "A Hundred Cases of Entire Excision of the Tongue," published in the British Medical Journal. However, Whitehead noted that he had first used his varnish over a decade earlier [9]. The original varnish comprised iodoform and turpentine, offering both antiseptic and anesthetic effects. The turpentine component notably halted capillary bleeding swiftly and reduced postoperative discomfort in many patients [9,10]. Today, Whitehead's varnish is synthesized using 10 g of iodoform, 10 g of benzoin, 7.5 g of prepared storax (a resinous exudate from the sweetgum tree), 5 g of balsam of Tolu (a resin obtained from South American balsam trees), and enough solvent ether to reach a total volume of 100 ml [10].

The evolution of iodoform's medical applications continued with Rutherford Morison's introduction of BIPP in 1916. In his article "The Treatment of Infected Suppurating War Wounds," published in The Lancet, Morison described the use of BIPP during World War I to treat gunshot wounds [11]. He reported accelerated healing and reduced infection rates when BIPP was applied to open wounds [5]. Morison's original formulation consisted of 16 oz of iodoform, 8 oz of bismuth subnitrate, and 8 oz of liquid paraffin mixed together in a mortar [11]. Today, BIPP is synthesized using similar ingredients but with more sophisticated methods, maintaining its role as a valuable wound-packing material in surgical procedures.

Modern forms of iodoform have diversified, finding applications across various medical fields. Alveogyl, for instance, is a brown fibrous paste containing iodoform commonly used in dentistry for the treatment of dry sockets. These developments underscore iodoform's enduring significance in surgery, where its antiseptic properties continue to be leveraged for improved patient outcomes. The historical advancements in iodoform formulations highlight their versatility and efficacy in surgical settings. Its incorporation into various antiseptic preparations has proven beneficial in general surgery, wound management, and postoperative care.

## Review

Surgical outcomes, clinical applications, and characteristics

Iodoform is utilized across various surgical specialties due to its potent antiseptic properties. Its applications span ear, nose, and throat (ENT) surgery, neurosurgery, oral and maxillofacial surgery, and general dental practice.

ENT Surgery

In ENT surgery, iodoform, often in the form of BIPP, is commonly used for dressing and cavity packing after procedures involving the facial skeleton, petrous bones, and sinuses. BIPP can be left in nasal and ear cavities for up to 10 days, providing sustained antiseptic effects [12]. It is frequently employed following the reduction of nasal fractures to prevent infection and in the management of epistaxis [13]. However, due to the risk of toxicity from enhanced absorption into the circulation, BIPP is not recommended for use on open wounds.

In myringoplasty procedures, BIPP has demonstrated success rates comparable to tri-adcortyl ointment for small and subtotal perforations, making it a viable option for tympanic membrane repair [14].

Neurosurgery

In neurosurgery, BIPP is primarily used for packing surgical sites post-transsphenoidal surgery, skull base operations, and treating chronic infective scalp wounds. Its effectiveness is attributed to several key properties: it renders impregnated gauze impervious to blood and bodily fluids, limiting bacterial nutrition; it stimulates granulation tissue growth; and it maintains stability even in the presence of necrotic tissue, facilitating clean wound management [8].

Notably, BIPP has shown promise in managing intraoperative cerebrospinal fluid (CSF) leakage during endoscopic transsphenoidal surgery. A retrospective review of 120 endoscopic pituitary operations performed between January 2002 and May 2008 reported a low incidence of postoperative CSF leakage (1.7%) when using BIPP, favorably compared with other methods [15]. The technique involves layering fibrin glue and gelatin sponge, followed by packing with BIPP in the sphenoid sinus. This approach minimizes further dissection, avoids non-absorbable foreign materials, and reduces postoperative CSF leakage. BIPP supports the repair construct, prevents irritation of exposed dura (which could lead to meningitis), and provides a substrate for fibroblasts during healing. Postoperative endoscopy typically reveals regrowth of sphenoid mucosa over the sellotomy, indicating successful healing [15].

Oral and Maxillofacial Surgery

Iodoform has wide-ranging applications in oral and maxillofacial surgery as an antiseptic material for various surgical interventions. Formulations such as BIPP, Whitehead’s varnish, Alveogyl, and Vitapex® are employed based on clinical needs [16].

Predominantly, iodoform is used following the surgical treatment of ameloblastomas and keratocystic odontogenic tumors. Post-resection, the cystic cavity is packed with iodoform-impregnated gauze, facilitating secondary healing. Its efficacy extends to managing dead spaces and cavities resulting from procedures like marsupialization, enucleation, and curettage of various cysts and tumors, as well as some fibro-osseous lesions of the jaws. The use of iodoform in these cavities not only promotes healing but also demonstrates its broad utility in treating abscesses, nasal cavities, and mastoid cavities [1].

The safety profile of iodoform and its derivatives, such as iodine powder-based dressing and Whitehead’s varnish, is commendable, with no reported complications following their application in jaw lesions. However, complications associated with BIPP, specifically related to toxicity, have been documented [1,17-19]. A systematic review indicated a high safety profile for BIPP in packing jaw lesions, with only two toxicity cases that resolved quickly upon removal of the packing [1].

General Dental Practice

In general dental practice, iodoform stands out for its antiseptic properties, whether used alone or mixed with eucalyptus oil or glycerine [16]. It's applied in the treatment of dry sockets, as a dressing for large cystic defects, and for soft tissue dressing in cases of wound dehiscence or to promote healing. Following tooth extractions, dental patients benefit from the use of iodoform packing strips and paste for alveolar osteitis in sockets, leading to pain relief and healing [20].

Metabolism, clearance, and radiographic appearance

Radiographic Appearance and Imaging Considerations

BIPP packs are radiopaque due to the high atomic number of bismuth (Z=83), making them readily identifiable on plain radiography (X-ray) [21]. This radiopacity is typically sufficient to confirm the presence and location of the pack within surgical cavities. However, in situations such as persistent epistaxis requiring interventional radiographic treatment, the high concentration of bismuth can cause complete opacification on radiographs. This interference necessitates the removal of the BIPP pack to allow for accurate imaging and effective intervention [1,21].

On computed tomography (CT) scans, BIPP exhibits extremely high attenuation values (>3000 Hounsfield units), leading to severe degradation of image quality due to streak artifacts [8]. These artifacts can obscure underlying anatomical details and pathology, complicating diagnostic evaluations. Similarly, on magnetic resonance imaging (MRI), BIPP packs have imaging characteristics similar to muscle tissue, which may result in misinterpretation if the radiologist is unaware of their presence.

**Figure 2:**
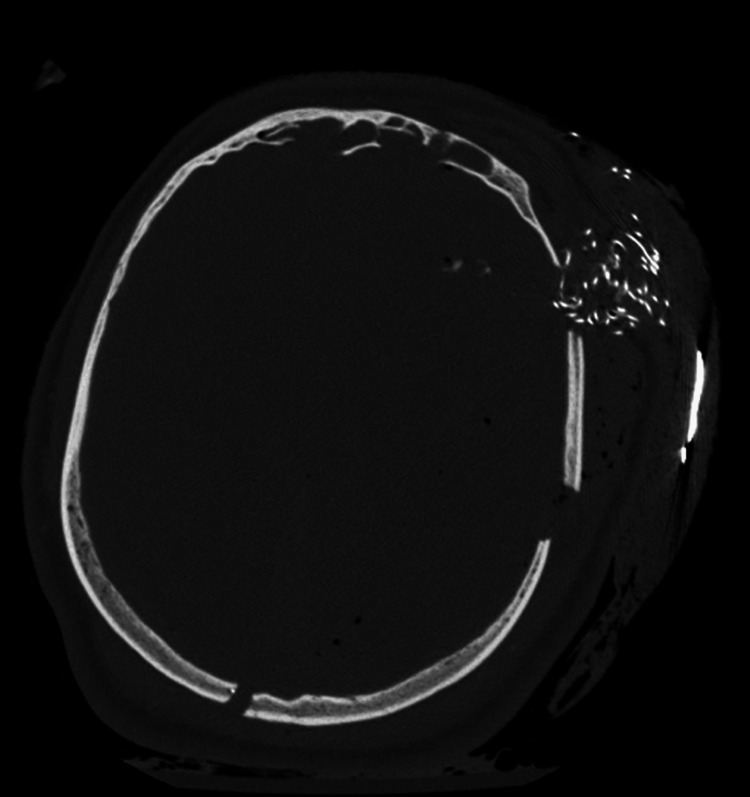
CT scan of the skull taken in an Axial (horizontal) view with BIPP packing incorporating radiopaque marker strips CT: computed tomography, BIPP: bismuth iodoform paraffin paste

To aid in the identification of BIPP packs, especially if dislodged or lost within cavities, marker strips are incorporated into the packs (Figure 2) [5]. These markers enhance visibility in imaging studies, facilitating accurate localization and reducing the risk of inadvertent retention.

Clinical Implications and Differential Diagnosis

It is crucial for healthcare providers to communicate the use of BIPP packs to the interpreting radiologist to prevent misdiagnosis. Residual iodoform packing can be mistakenly interpreted as infection, tissue necrosis, hematomas, or ongoing bleeding, particularly on postoperative imaging. Such misinterpretations may lead to unwarranted surgical interventions or additional diagnostic procedures.

The radiopacity of BIPP also has implications for interventional procedures. For example, before performing embolization for nasal epistaxis, removal of the BIPP pack is necessary because its presence can interfere with imaging guidance and treatment efficacy [1].

Although rare, bismuth encephalopathy can occur due to absorption of bismuth from BIPP packing [8]. On CT imaging, bismuth neurotoxicity manifests as areas of diffuse hypodensity in the gray matter, with patchy areas of high attenuation in the basal ganglia and cerebral cortex, representing concentrations of bismuth within neural tissue. The low-attenuation areas reflect cerebral edema, which often occurs adjacent to the BIPP pack.

MRI may reveal more prominent findings, such as a stippled appearance in the deep white matter [8]. If signs of bismuth toxicity are present, prompt removal of the BIPP pack can lead to rapid resolution of symptoms [1]. Continued monitoring and appropriate workup are essential to prevent further neurological complications.

Complications

While iodoform and its formulations are widely used for their antiseptic properties in surgical practice, several potential complications can arise from their use. These complications range from toxicities and allergic reactions to mechanical issues and considerations during pregnancy. Awareness of these potential adverse effects is crucial for healthcare providers to ensure patient safety and optimal outcomes.

Although BIPP is generally safe, absorption of bismuth subnitrate from the packing can occasionally occur, potentially leading to neurotoxicity by interfering with oxidative cerebral metabolism. Symptoms of bismuth toxicity may include neurological symptoms, including headache, confusion, ataxia, myoclonus, and, in rare cases, encephalopathy, and gastrointestinal symptoms such as nausea and stomatitis. These symptoms have been documented in the literature [5,8]. Historically, bismuth toxicity was more common when BIPP was used extensively for large wounds, such as gunshot injuries, during the First World War [22]. In modern medical practice, instances are rare but have been reported, particularly when large amounts of BIPP are applied. For example, cases of severe bismuth toxicity have been detailed following the use of BIPP gauze after total maxillectomy procedures [23,24]. In both cases, the removal of the gauze led to patient recovery, highlighting the importance of the cautious application of BIPP in large quantities. Prompt recognition of symptoms and immediate removal of the BIPP packing are essential steps in managing bismuth toxicity. Recovery is typically rapid once the source of bismuth exposure is eliminated.

Iodoform can be toxic in high quantities due to its metabolism into diiodine oxide and the production of carbon monoxide, both hazardous substances for the human body [25]. It is often challenging to distinguish between symptoms directly attributed to iodoform, iodine release, or carbon monoxide production. Symptoms of iodoform and iodine toxicity include neuropsychiatric effects such as general malaise, reduced appetite, headaches, restlessness, depressive states or confusion, lethargy, and light coma; gastrointestinal issues like nausea and vomiting; cardiovascular symptoms including fever and rapid heartbeat; and iodine-specific symptoms such as skin eruptions, metabolic acidosis, kidney failure, decreased white blood cell count, and disruptions in thyroid and liver function [17-19,26,27]. Elevated iodine levels in the body are indicative of iodoform toxicity, and since over 90% of ingested iodine is expelled through urine, measuring urinary iodine can serve as a marker for exposure [28]. Symptoms resulting from carbon monoxide production during iodoform metabolism include flu-like symptoms such as headache, dizziness, weakness, upset stomach, vomiting, chest pain, and confusion.

Iodoform toxicity was more frequent in the past when BIPP was used extensively for large wounds. Contemporary cases have been documented, such as severe toxicity following the use of BIPP in maxillofacial surgery [23,24]. Removal of the iodoform-containing material led to patient recovery. Awareness and prompt recognition of symptoms are crucial. Removal of the iodoform-containing material is essential, and supportive care should be provided as needed.

Allergic reactions to BIPP are relatively rare, affecting about 1% of patients, though the incidence rises to 12% among individuals previously exposed to BIPP [6,29]. Retrospective studies have reported an allergy risk between 0.4% and 6% [30,31]. Clinical manifestations of BIPP allergy include local reactions, such as inflammation at the application site and atopic responses, as well as systemic symptoms like fatigue, confusion, depression, memory loss, aggressive behavior, paranoid ideation, and suicidal thoughts. A prospective patch-testing study identified iodoform, rather than iodine, as the allergenic component in BIPP, a finding that is crucial for guiding management and preventive strategies [29]. To mitigate the risk of allergic reactions, patch testing is recommended for patients with a history of BIPP exposure prior to further surgery, while it is discouraged in those without prior exposure due to the low allergy incidence and the potential risk of sensitization. Complications from allergic reactions to BIPP can include residual perforation following myringoplasty, indicating risks to healing and tissue integrity [31]. Additionally, a unique case of delayed postoperative facial nerve palsy has been reported in association with BIPP allergy, likely due to local inflammatory responses rather than inherent neurotoxicity, as studies applying BIPP directly to nerves in animal models showed no impairment, suggesting that neurological symptoms may arise from allergic inflammation rather than direct toxicity [32].

Exposure to iodoform can lead to dermatological side effects, particularly in individuals susceptible to conditions like dermatitis herpetiformis (DH). A case report documented a significant DH flare in a 19-year-old woman with a controlled six-year history of DH after iodoform packing strips were used during a dental procedure for alveolar osteitis [20]. Following the procedure, she experienced an intense eruption of pruritic papules and vesicles on her shoulders, elbows, and knees, and symptoms more severe than her typical DH exacerbations. Removing the iodoform packing strips led to a resolution of her skin lesions and symptoms, indicating a strong temporal association. Products containing iodine are known to trigger DH flares, especially when applied to non-keratinized mucosal surfaces where systemic absorption is higher. Therefore, caution is recommended when using iodine-containing products in patients with a history of DH to prevent potential flare-ups.

Mechanical complications associated with iodoform use in surgical packing can significantly impact healing and patient comfort. Common issues include inflammation at the mucosal opening, causing discomfort and delayed recovery, as well as dislodgement of packing material into the cavity, complicating the healing process. Premature soft tissue closure of an intended drainage window can further impede healing and lead to recurrence, while secondary infections may arise from iodoform residues and inadequate irrigation [33]. Contributing factors include inadequate surgical technique, such as incomplete unroofing of lesions, which can result in the recurrence of the condition being treated. Preventive strategies include using obturators to stabilize packing material and maintain drainage patency, employing thorough surgical techniques to reduce recurrence risk, and ensuring adequate postoperative care with irrigation and close monitoring to prevent infections. Healthcare providers should remain vigilant for signs of these mechanical complications and address them promptly to support optimal healing.

The use of iodine-containing compounds, such as iodoform and BIPP, during pregnancy raises concerns about iodine transfer to the fetus and potential impacts on thyroid function, as iodine can cross the placenta and equilibrate with iodide [34]. This poses a theoretical risk of fetal thyroid dysfunction, where excessive maternal iodine intake could induce fetal hyperthyroidism or hypothyroidism, with severe cases potentially leading to congenital iodide goiter, tracheal compression, or even death [35,36]. Additionally, maternal hyperthyroidism could be exacerbated by exposure to iodine-containing compounds, potentially triggering a thyrotoxic crisis, though no incidents have been directly linked to BIPP usage [12]. While short-term iodine exposure, such as pre-surgical preparation, is generally safe and rarely causes fetal issues, cases of fetal hypothyroidism have been documented following repeated maternal topical iodine exposure, underscoring the need for caution [35,37]. Given these considerations, it is advised to exercise caution when using iodoform-containing products in pregnant patients or those with thyroid disorders, with vigilant monitoring to minimize potential risks.

## Conclusions

Iodoform, in its various forms such as BIPP, Whitehead's varnish, and Alveogyl, has proven to be an effective antiseptic and wound-healing agent across multiple surgical fields, including ENT neurosurgery, oral and maxillofacial surgery, and dentistry. Its unique properties facilitate chemical debridement, stimulate granulation tissue, and provide both antiseptic and anesthetic effects, making it a valuable asset for managing surgical wounds and cavities. Despite its benefits, careful consideration must be given to potential toxicity and allergic reactions, particularly with prolonged use or in patients with prior sensitivities. The comprehensive review of iodoform's applications and safety profiles underscores its continued relevance and utility in modern surgical practices while also highlighting areas for future research to optimize its use and minimize risks.
